# Reduction of Chronic Fatigue Through Transcranial Direct Current Stimulation and Low-Intensity Aerobic Exercise: A Case Study of Two Patients With Granulomatosis With Polyangiitis

**DOI:** 10.7759/cureus.74105

**Published:** 2024-11-20

**Authors:** Alexandre M Dos Santos, Vanessa P De Andrade, Paulo Roberto S Silva, Marcus V Grecco, Julia Maria D'Andrea Greve, Samuel K Shinjo

**Affiliations:** 1 Rheumatology, Faculdade de Medicina da Universidade de São Paulo (FMUSP), Sao Paulo, BRA; 2 Movement Studies Laboratory, Institute of Orthopedics and Traumatology, Hospital das Clínicas, Faculdade de Medicina da Universidade de São Paulo (FMUSP), Sao Paulo, BRA

**Keywords:** aerobic exercises, anca-associated vasculitis, chronic fatigue, granulomatosis with polyangiitis, neuromodulation

## Abstract

Granulomatosis with polyangiitis (GPA) is a systemic vasculitis that can lead to persistent pain and fatigue, significantly impacting patients' quality of life. This study assessed the effects of transcranial direct current stimulation (tDCS) combined with aerobic exercise as a non-pharmacological intervention for managing fatigue in GPA patients. Two patients were randomly assigned to receive either active tDCS or simulated tDCS stimulation (sham) during low-intensity aerobic exercise. The assessments included body mass index, fat and lean mass percentages, waist-to-hip ratio, C-reactive protein, erythrocyte sedimentation rate, and disease activity using the Birmingham Vasculitis Activity Score. Chronic fatigue was measured using the Modified Fatigue Impact Scale, the Fatigue Severity Scale, and the visual analog scale for fatigue. Sleep quality, activities of daily living, and functional capacity were evaluated through standardized tests. Results indicated that tDCS significantly reduced chronic fatigue by approximately 60%, nearly twice as much as the sham patient. Additionally, the tDCS patient showed improvements in physical activity levels, functional capacity, handgrip strength, daily activities, and sleep quality. In contrast, the sham patient showed declines in physical activity and minimal improvement in chronic fatigue. Overall, tDCS appears to be a promising intervention to enhance chronic fatigue and the quality of life in patients with GPA without causing disease reactivation or adverse effects.

## Introduction

Granulomatosis with polyangiitis (GPA) is a primary systemic vasculitis that primarily affects small- and medium-sized vessels and is characterized by granulomatous and necrotizing inflammation, predominantly involving the lungs and kidneys [1,2]. Additionally, patients often experience pain and fatigue, which may persist even without active disease [3,4], leading to a decline in quality of life and the ability to perform activities of daily living [5,6].

Transcranial direct current stimulation (tDCS) combined with aerobic exercise has emerged as a non-pharmacological alternative for the treatment of pain, particularly fatigue, in patients with rheumatic disease. This therapy is both safe and promising, as evidenced by preliminary studies from Andrade et al. [7,8], which showed a sustained reduction in fatigue that persisted even after 60 days.

In a pioneering approach to GPA, our study aimed to evaluate the effects of combined low-intensity aerobic exercise and tDCS on the treatment of chronic fatigue, with a secondary objective of assessing the safety of this innovative therapy.

## Case presentation

Two patients with GPA were included, as defined by the 2022 American College of Rheumatology/European League Against Rheumatism (ACR/EULAR) criteria [2]. The patient did not meet the classification criteria for fibromyalgia established by the ACR/EULAR, such as the presence of widespread pain in specific tender points, along with other clinical requirements necessary for the diagnosis of the condition [9]. Both patients provided written informed consent before participation in the study.

We conducted evaluations at three time points: one week before the intervention (pre-sessions), midway through the sessions (after five sessions), and one week following the sessions (post-10 sessions).

Parameters assessed pre- and post-session/intervention included age, body mass index (BMI), body composition via bioelectrical impedance (lean and fat mass percentages), waist-to-hip ratio, acute phase reactants (C-reactive protein and erythrocyte sedimentation rate), and disease activity measured by the Birmingham Vasculitis Activity Score (BVAS) [10].

Additionally, we evaluated the following parameters at three time points (pre-intervention, after five sessions, and post-intervention): chronic fatigue using the Modified Fatigue Impact Scale (MFIS) [11] (range: 0-84), where scores above 38 indicate chronic fatigue; the Fatigue Severity Scale (FSS) [12] (range: 9-63), with scores above 36 indicating fatigue; and the visual analog scale of fatigue (VAS fatigue) (range: 0-10). We also assessed sleep quality using the Pittsburgh Sleep Quality Index (PSQI) [13], activities of daily living using the Health Assessment Questionnaire (HAQ) [14] (range: 0.00-3.00), functional capacity with the sit-to-stand test [15], upper extremity strength using the handgrip test [16], and the metabolic equivalent (MET) values in the IPAQ-SF are calculated by assigning specific values to walk (3.3 METs), moderate-intensity (4.0 METs), and vigorous-intensity activities (8.0 METs). Total MET minutes per week are obtained by multiplying the duration and frequency of each activity by its respective MET value [17].

The patients were selected and randomly assigned using software (R software, version 4.4.1 for Windows, R Foundation for Statistical Computing, Vienna, Austria, https://www.R-project.org/)) under double-blind conditions to receive either active tDCS or simulated tDCS stimulation (sham). Both patients participated in concurrent low-intensity aerobic exercise performed simultaneously with either tDCS or a sham intervention, determined by cardiopulmonary exercise testing on a treadmill, where we used the heart rate at ventilatory threshold 1 as a reference, and the training was conducted below this threshold, ensuring low intensity.

The positioning of the tDCS electrodes was applied with the anode placed at C3 and the cathode at Fp2, following the International 10-20 EEG System, as previously described in studies conducted by our research group [8,18].

**Figure 1:**
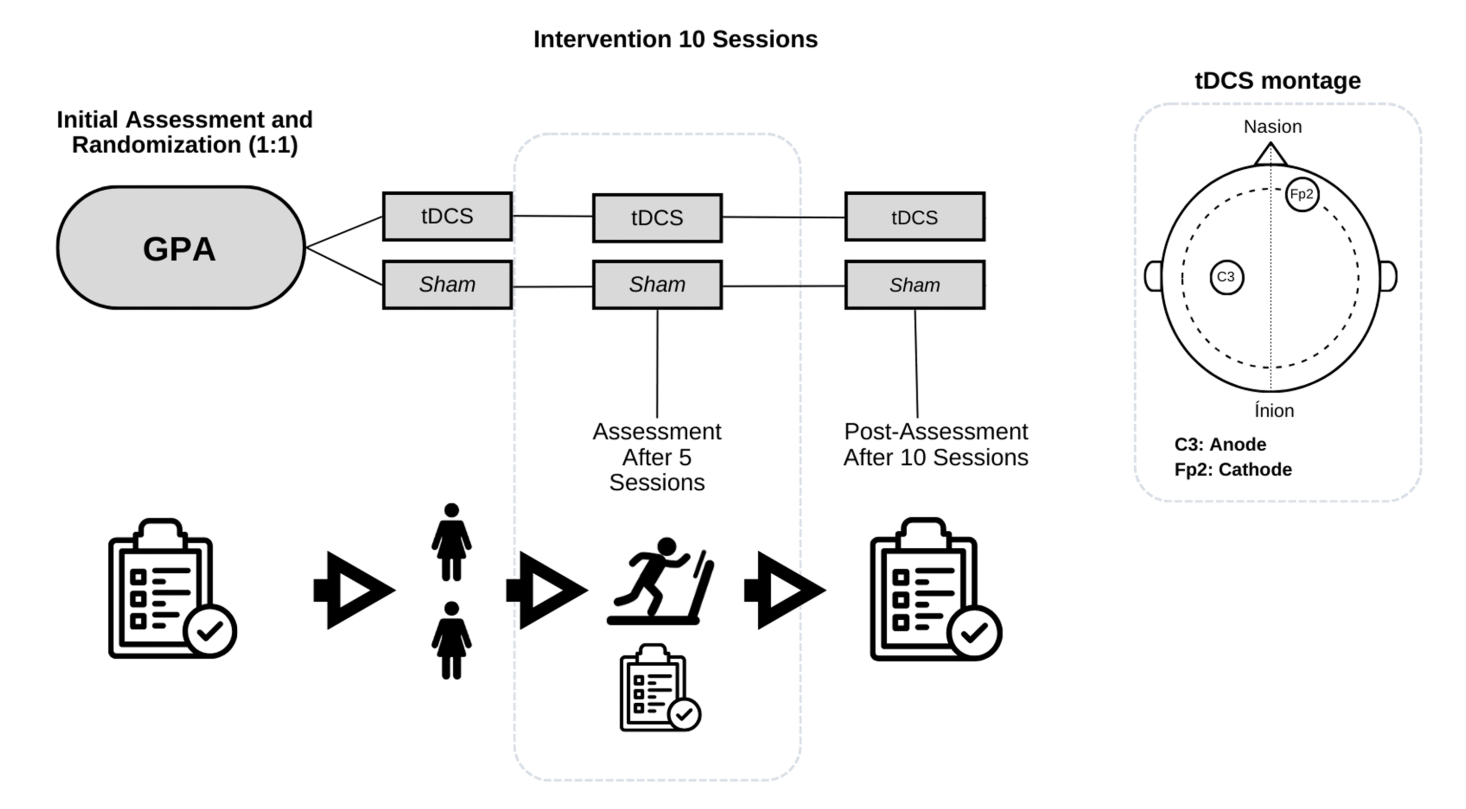
Flowchart of tests, assessments, and intervention GPA: granulomatosis with polyangiitis; sham: simulated tDCS stimulation; tDCS: transcranial direct current stimulation Image Credit: Authors

Both patients underwent a similar protocol, differing only in the specifics of the intervention. The patient receiving active tDCS was administered a continuous 2 mA current for 20 minutes, while the sham group received a 2 mA ramp-down stimulus for 30 seconds to mimic the active procedure. Concomitantly, each session included 30 minutes of aerobic training: a five-minute warm-up, 20 minutes of low-intensity exercise, and a five-minute cooldown, all supervised by a physical education professional. Sessions were conducted daily in the morning (Monday to Friday) over two consecutive weeks, totaling 10 sessions for each patient. Before each session, we assessed potential side effects and measured the region on the scalp for electrode placement, ensuring a consistent setup across both groups (Figure 1).

Due to the small sample size (n=2) and the division into two interventions, descriptive statistics were not calculated. Instead, individual data for each patient were presented in absolute values, with qualitative variables expressed as relative (%) and absolute (n) frequencies. We compared the time points (baseline, after five sessions, and after 10 sessions) by calculating the percentage variation for each patient individually, as shown in the following equation.

\( \text{Percentage variation} = \left( \frac{\text{post value} - \text{pre value}}{\text{pre value}} \right) \times 100 \)

Additionally, we used a spider/radar chart for visual comparison, graphically representing the variables' variation over time [19]. All statistical analyses and graphs were generated using R software.

Case 1: sham patient

The 57-year-old white female patient, with a high school education and a BMI of 26.5 kg/m², presented with no active GPA, as indicated by a BVAS score of 0. Her resting blood pressure was 120/70 mmHg, and her heart rate was 73 bpm. Body composition analysis revealed 41.0% body fat, 31.7% lean body mass (19.4 kg), and a waist-to-hip ratio of 0.91. Regarding her medication for GPA, the patient was on prednisone (5 mg/day) and rituximab. Additionally, she had a prior diagnosis of anxiety disorder, as reported by the patient, and was managing it with fluoxetine (20 mg daily). The patient reported chronic fatigue for at least six months, which significantly impacted her ability to perform daily activities.

At the time of her diagnosis, the patient presented with chronic sinusitis, polyarthritis, purpura and ulcers on the lower limbs, right hearing loss, glomerulonephritis, positive cytoplasmic ANCA, and positive anti-serine proteinase.

Physical activity levels indicated MET scores of 693, 655, and 160 MET-min/week at pre-sessions, after five sessions, and post-10 sessions, respectively. These correspond to a 5.5% decrease from pre-sessions to after five sessions, a 75.6% reduction from after five sessions to post-10 sessions, and an overall decline of 76.9% from pre-sessions to post-10 sessions.

Sedentary time decreased from 5.5 hours at pre-sessions to 3.5 hours after five sessions and 3.0 hours post-10 sessions, representing a 35.5% reduction between pre-sessions and after five sessions, a 15.4% improvement between after five sessions and post-10 sessions, and an overall decrease of 45.4% from pre-sessions to post-10 sessions.

Functional capacity decreased from 15 repetitions at pre-sessions to 12 repetitions at post-10 sessions, representing a 20% reduction in performance. The timed up-and-go test showed improvement, with times of 6.9 seconds at pre-sessions and 6.3 seconds at post-10 sessions, reflecting an 8.1% improvement. Handgrip strength remained stable at 22 kgf in both pre- and post-10 sessions.

The HAQ scores were 0.78, 0.67, and 0.78 at pre-sessions, after five sessions, and post-10 sessions, indicating a 14.1% improvement between pre-sessions and after five sessions, followed by a 16.4% decline between after five sessions and post-10 sessions, resulting in no overall change in daily living activities.

Sleep quality showed scores of 7.0, 5.0, and 7.0 at pre-sessions, after five sessions, and post-10 sessions, respectively. While an improvement was observed between pre-sessions and after five sessions, a 40% worsening occurred after five to post-10 sessions, leading to no overall difference between pre- and post-10 session scores.

Fatigue was evaluated using three different metrics at three time points. Specifically, the fatigue VAS scores were 6.0, 4.0, and 4.0 at pre-sessions, after five sessions, and post-10 sessions. A 33.3% improvement was noted between pre-sessions and after five sessions, with no further change at post-10 sessions, maintaining an overall improvement of 33.3%.

The initial FSS score was 49.0 points, indicating the presence of chronic fatigue (i.e., ≥ 36 points), followed by scores of 33.0 and 31.0 points, showing a 32.6% improvement from baseline to after five sessions, with an additional 6.0% improvement after 10 sessions, resulting in an overall enhancement of 36.7%.

**Figure 2:**
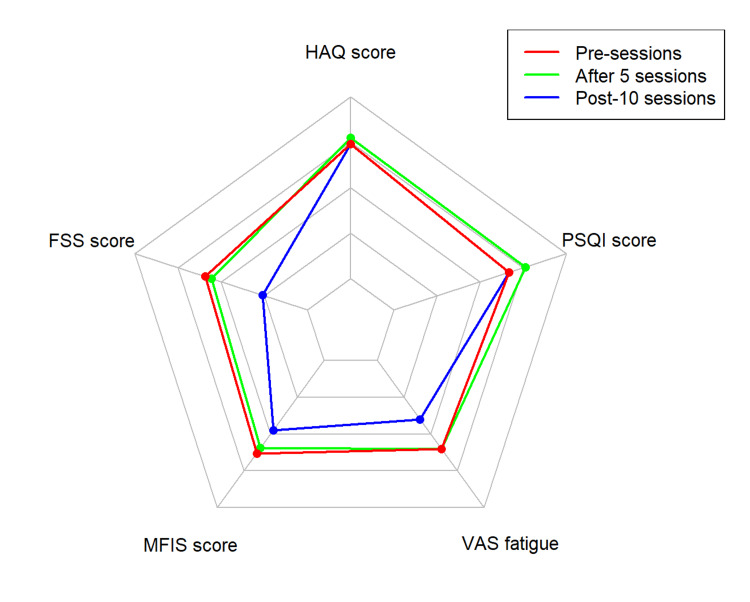
Radar chart of fatigue, daily activities, and sleep quality of sham patient FSS: Fatigue Severity Score; HAQ: Health Assessment Questionnaire; MFIS: Modified Fatigue Impact Scale; PSQI: Pittsburgh Sleep Quality Index; VAS: visual analog scale, sham: simulated tDCS stimulation Image Credit: Authors

The initial MFIS score was 44.0, confirming the presence of fatigue pre-intervention (i.e., ≥ 38 points), followed by scores of 34.0 and 31.0 at pre-sessions, after five sessions, and post-10 sessions, respectively. These values represent a 22.7% improvement after five sessions, an additional 8.8% improvement after 10 sessions, and a total improvement of 29.5% (Figure 2). The patient did not experience any adverse effects or disease relapses.

Case 2: tDCS patient

A 42-year-old female with a high school education presented with no active GPA, as reflected by a BVAS of 0. Inflammatory markers included a CRP level of 0.5 mg/L and an ESR of 7.0 mm/h. She had a BMI of 23.4 kg/m² and a waist-to-hip ratio of 0.84. Body composition analysis revealed 35.2% body fat and 34.9% lean mass (20.4 kg). Her systemic blood pressure was 130/90 mmHg, and her resting heart rate was 69 bpm. The patient also had prediabetes, managed with metformin, and was receiving prednisone at 20 mg/day, with ongoing outpatient tapering, alongside rituximab for GPA management. Additionally, the patient reported chronic fatigue for at least six months, which, among other factors, worsened her quality of life. At the time of diagnosis, she had sinusitis, hearing loss, lacrimal canal occlusion, pneumonia, and positive cytoplasmic ANCA.

Pre-sessions, the patient’s weekly MET score was 320 MET min/week, which increased to 933 after five sessions and 1,626 post-10 sessions. This represents an improvement of 191.6% between pre-sessions and after five sessions, a 74.3% increase between five sessions and post-10 sessions, and a total increase of 408.1%. Sedentary time has decreased from 6.5 hours at pre-sessions to six hours after five sessions and 4.0 hours post-10 sessions, reflecting reductions of 7.7%, 33.3%, and 38.5%, respectively, from pre-sessions to post-10 sessions.

To evaluate functional capacity, assessments were conducted at two time points: pre-sessions and post-10 sessions and after 10 sessions. The sit-to-stand test demonstrated an increase from 12 repetitions pre-sessions to 14 repetitions post-10 sessions, representing a 16.7% improvement in performance. The timed up-and-go test also showed enhancement, with times of 6.4 seconds pre-sessions and 6.3 seconds post-10 sessions, reflecting a 2.8% improvement. The handgrip test showed a pre-session strength of 22 kgf, which increased to 25 kgf post-10 sessions, indicating a 13.6% increase.

The HAQ scores were 0.33, 0.22, and 0.00 at pre-sessions, after five sessions, and post-10 sessions, respectively. This reflects a 33.3% improvement between pre-sessions and after five sessions, followed by a decline to the minimum score post-10 sessions, demonstrating sustained improvement at minimal levels throughout the entire protocol.

Sleep quality scores were 9.0, 2.0, and 2.0 at pre-sessions, after five sessions, and post-10 sessions, respectively. While an improvement of approximately 78% was observed between pre-sessions and after five sessions, the scores remained constant from after five sessions to post-10 sessions, resulting in an overall enhancement of 78% between pre-sessions and post-10 session scores.

The fatigue VAS scores were 8.0, 6.0, and 3.0, at pre-sessions, after five sessions, and post-10 sessions, respectively. A 25.0% improvement was noted between pre-sessions and after five sessions, followed by a 50.0% enhancement from after five sessions to post-10 sessions, resulting in an overall improvement of 62.5%.

Additionally, fatigue was assessed using the FSS score, with an initial value of 52.0, indicating the presence of chronic fatigue, followed by scores of 35.0 and 23.0 at pre-sessions, after five sessions, and post-10 sessions, respectively. This represents a 32.7% improvement between pre-sessions and after five sessions, followed by an additional 34.3% enhancement post-10 sessions, resulting in a total improvement of 55.7%.

**Figure 3:**
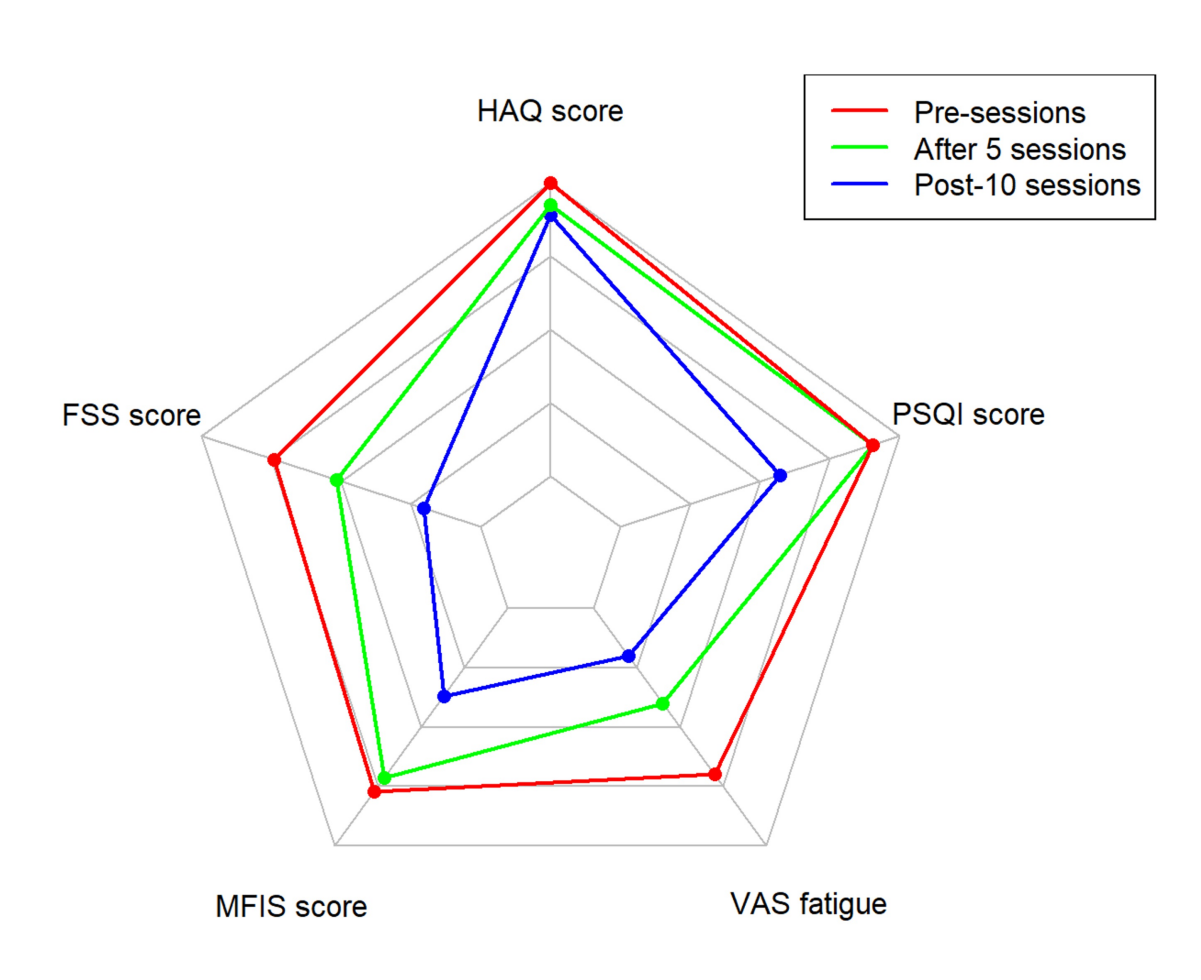
Radar chart of fatigue, daily activities, and sleep quality of tDCS patient FSS: Fatigue Severity Score; HAQ: Health Assessment Questionnaire; MFIS: Modified Fatigue Impact Scale; PSQI: Pittsburgh Sleep Quality Index; VAS: visual analog scale, tDCS: transcranial direct current stimulation Image Credit: Authors

The MFIS score was 53 points, confirming the presence of fatigue pre-intervention. However, it decreased to 24 points and 19 points at pre-sessions, after five sessions, and post-10 sessions, respectively, reflecting a 54.7% improvement after five sessions, followed by an additional 20.8% improvement post-10 sessions, resulting in a total enhancement of 64.1% (Figure 3). No adverse events or disease relapses were reported in the patient.

## Discussion

Patients with GPA frequently experience chronic fatigue and heightened physical inactivity, often independently of sedentary behavior [20]. Emerging evidence suggests that physical activity and structured exercise can alleviate symptoms such as chronic fatigue and chronic pain, enhancing the overall quality of life for ANCA-associated vasculitis [21,22].

In our study, two patients displayed significant fatigue and inactivity. Both sham and tDCS treatments resulted in notable fatigue reductions. However, the sham patient primarily benefited from aerobic exercise, a well-established intervention for managing fatigue and chronic pain, likely influenced by the placebo effect through central nervous system modulation [23,24].

In contrast, the patient undergoing tDCS combined with low-intensity aerobic exercise exhibited nearly double the reduction in fatigue, accompanied by improvements in functional capacity and activities of daily living. This amplified response could be attributed to central mechanisms, including central sensitization [25]. Future studies incorporating functional magnetic resonance imaging of the motor cortex may provide deeper insights into the neural adaptations associated with aerobic exercise and the combined tDCS intervention.

Despite the limitations of our small sample size, these findings underscore the potential of targeted interventions in specific neural regions to yield significant health benefits. Notably, patients reported improvements after just five sessions of tDCS, with continued enhancements through ten sessions; however, the dose-response relationship remains poorly characterized in the literature. Studies from our group indicate sustained fatigue reductions within the tDCS cohort, utilizing similar protocols [7,8].

Chronic fatigue lasting beyond six months, even without active disease, presents a considerable therapeutic challenge in GPA management. Low-intensity aerobic exercise, such as conventional walking, may be an effective initial strategy to address this common symptom in ANCA-associated vasculitis. It is vital to promote increased physical activity to improve quality of life and mitigate cardiovascular risks in systemic vasculitis patients, including those with GPA [20]. Rheumatologists should actively encourage this practice and facilitate referrals to exercise specialists for tailored exercise prescriptions.

Advances in technology and neuroscience have positioned tDCS as a promising adjunctive treatment for chronic fatigue, especially when combined with aerobic exercise. By targeting central mechanisms like central sensitization, tDCS enhances the benefits of physical activity, reducing fatigue and improving functional capacity and quality of life [26]. This approach is particularly valuable for conditions such as GPA, where fatigue persists despite traditional treatments. Further research will help refine its use and assess its broader clinical impact.

## Conclusions

Notably, there was a remarkable reduction in chronic fatigue of approximately 60% following tDCS, nearly double that observed in the sham patient. Our data also indicate significant enhancements in MET levels, suggesting increased physical activity. These findings emphasize that tDCS may be particularly effective in alleviating chronic fatigue. Additionally, improvements were seen in functional capacity, handgrip strength, activities of daily living (by HAQ), and sleep quality. In contrast, the sham group experienced a decline in physical activity and only a 30% improvement in chronic fatigue, with no reduction in sedentary time. In summary, our findings suggest that tDCS may be a promising strategy, among other multidisciplinary approaches, to help improve the quality of life and fatigue in patients with GPA.
